# Pesticides and Health Effects: Karpati et al. Respond

**Published:** 2005-03

**Authors:** Adam Karpati, Mary C. Perrin, Jessica Leighton, Tom Matte, Joel Schwartz, R. Graham Barr

**Affiliations:** New York City Department of Health and Mental Hygiene, New York, New York, E-mail: akarpati@health.nyc.gov; Division of Environmental Health, Harvard School of Public Health, Boston, Massachusetts; Department of Medicine, Columbia University Medical Center, New York, New York

In her letter, Ziem raises the issue of exacerbations of respiratory illness and other health effects of pesticide exposure that we did not measure in our study (Karpati et al. 2004). Our analysis was designed to evaluate only whether a population-level effect on emergency department visits, specifically on asthma and other respiratory illnesses, was evident following pyrethroid pesticide spraying. Similar study designs, despite their limitations, have proven to be sensitive methods of identifying population-level health impacts from exposure to criteria air pollutants from exposure to unusual events, such as smoke from forest fires. Moreover, our analysis did identify adverse population-level health effects of elevated ozone and particulates. As we noted in our discussion, the results of the analysis for pesticide exposure do not rule out the possibility that certain individuals might have been affected by exposure to the agent. Also, our focus was on emergency department visits, which generally signify more serious illness, although in urban neighborhoods even milder illnesses are often treated in such settings. However, if, in fact, certain individuals experienced asthma exacerbations following exposure, we believe our study demonstrates that their number was small enough that it did not result in a population-level increase in emergency department visits for asthma and chronic obstructive pulmonary disease.

In our analysis we evaluated only respiratory complications of pesticide spraying to control West Nile virus, and we did not purport to measure possible neurotoxic or other nonrespiratory effects. Also, we did not evaluate the efficacy of pesticide spraying for mosquito control or its cost–benefit ratio with regard to pesticide-related health effects.
